# Suppressing immunotherapy by organ-specific tumor microenvironments: what is in the brain?

**DOI:** 10.1186/s13578-019-0349-0

**Published:** 2019-10-07

**Authors:** Chenyu Zhang, Dihua Yu

**Affiliations:** Department of Molecular and Cellular Oncology, The University of Texas M.D. Anderson Cancer Center, Houston, TX USA

**Keywords:** Immunotherapy, Therapeutic response, Tumor microenvironment, Brain metastasis

## Abstract

Recent breakthroughs in cancer immunotherapy have led to curative efficacy and significantly prolonged survival in a subset of patients of multiple cancer types; and immunotherapy has become the newest pillar of cancer treatment in addition to surgery, chemotherapy, radiotherapy and precision targeted therapies. In the metastatic disease setting, responses to immunotherapy are heterogeneous depending on the metastatic organ sites. The tissue-specific immuno-biology in the tumor microenvironments (TMEs) contributes to the differential therapeutic responses. Herein, we review the impact of tissue-specific tumor microenvironment on the efficacy of immunotherapy, with a focus on historically under-represented central nervous system (CNS) metastasis, which was excluded from most clinical trials. Retrospective examination of patient specimens and prospective clinical studies with immune checkpoint blockade (ICB) have established that brain can harbor an “active” immune microenvironment for effective immunotherapy. Regulation by the innate immune microglial cells and remodeling of the blood–brain barrier (BBB) may contribute to immunotherapeutic responses mediated by T lymphocytes. How to convert an “inactive” (cold) brain microenvironment into an “active” (hot) brain TME should be the focus of future efforts. Thus, procurement and complete examination of clinical specimens from brain metastases as well as development of appropriate preclinical brain metastasis models susceptible to external manipulation of the TME are critical steps towards that goal. A deeper understanding of the immuno-biology in distinct organ microenvironments will help to expand the benefits of immunotherapy to more needed patients.

## Background: immunotherapy

The idea of immunotherapy was conceptualized more than 100 years ago when German pathologist Rudolf Virchow first described the involvement of immune cells in human tumors [1], followed by American surgeon William Coley’s successful attempt to treat cancer patients by inoculating them with “Coley’s toxins”, a preparation of live bacteria that activates cancer patients’ immune system [2]. Even though in the ensuing century Coley’s approach was proven inconsistent and remained controversial, as proof of concept it still established that the human immune system can be utilized to attack cancer cells. Coley is thus widely recognized as the “Father of Cancer Immunotherapy” [3].

Beginning in the 1980s, therapeutic monoclonal antibodies, a bioengineered version of the naturally secreted immune molecule, emerged as a versatile platform of therapeutic agents against cancer [4, 5]. After more than 30 years of development, it has firmly established as a major modality of pharmaceutical agents as > 60 of monoclonal antibodies have been FDA approved for treatment of various human diseases [6]. During the same period another immunotherapy agent, the natural cytokine molecule interleukin-2 (IL-2), emerged as a promising anti-cancer agent and its recombinant form aldesleukin won FDA approval for treating metastatic renal cancer in 1992 [7]. IL-2 is an extremely potent and pleiotropic regulator of white blood cell (lymphocyte) activation and key functions of the immune system [8]. However, due to severe side effects, IL-2 has a narrow therapeutic window and its usage is limited only to treating selective advanced melanoma and renal cancer patients [9, 10].

It has long been known that CD8+ effector T cells have cytolytic capability that kills cancer cells [11]. In-depth understanding of the T cell biology took off in the 1980–1990s, which included the discovery of T cell receptors (TCR) [12, 13], identification of positive [14] and negative co-stimulatory molecules [15]. It was then postulated and demonstrated that appropriate manipulation of T cells may exert powerful anti-tumor activity in animal models [16]. After years of preclinical and clinical development, two forms of T cell-based immunotherapy, immune checkpoint blockade (ICB) and adoptive cell transfer (ACT) that includes chimeric antigen receptor T cell (CAR-T) therapy, have shown remarkable clinical efficacy in treating a wide range of advanced cancers [17–19]. These unprecedented results have propelled immunotherapy as the newest modality of cancer treatment in addition to other available therapies [20]. Currently both modalities of T cell-based immunotherapy are the focus of intensive research and clinical development in order to expand efficacy into more cancer types and frontline patient cohorts. While ICB has won FDA approval in a wide range of both solid and liquid cancer types [21], so far ACT therapies have been successful only in hematological malignancies [22]; hence, the current review will focus on ICB therapies because extensive organ-specific interactions between cancer cell and tumor microenvironment (TME) take place primarily in solid tumor metastases.

## Impact of organ-specific tumor microenvironments on immunotherapeutic responses

Different cancer types tend to colonize specific organ sites, as depicted by the “seed and soil” hypothesis of metastasis [23]. Many of the organ sites have distinctive immune microenvironments typified by the presence of distinct tissue-resident innate immune cells [24], such as osteoclasts in the bone, microglia in the brain, Kupffer cells in the liver, alveolar macrophages in the lung and peritoneal macrophages in the omentum (Table 1). These cells serve as frontline mediators of immune surveillance and local inflammation, as well as an important component of tumor-associated macrophage (TAM). Hence it is not surprising that the tissue-dependent difference is most important in response to immunotherapy.

**Table 1:** Tissue-resident innate immune cells in common organ sites of metastasis

Metastasis target organ	Innate immune cell type	Function and contribution
Bone	Osteoclasts	Multinucleated cells transformed from monocytes that breaks down and absorbs the bone tissue, critical in the bone homeostasis
Brain	Microglia	Myeloid lineage glial cells accounting for 10-15% of all cells in the brain, mediating immune surveillance and inflammation in homeostasis and diseases of the central nervous system
Liver	Kupffer cells	Specialized macrophages lining the walls of the liver sinusoids, serving as the primary clearing cell for critical metabolic and detoxification functions of the liver
Lung	Alveolar macrophages	High activity macrophages located in pulmonary alveoli, the terminal units of gaseous exchange, primarily responsible for removing respiratory dust and pathogens
Omentum	Peritoneal macrophages	Omental milky spot-located macrophage cells playing house-keeping roles in immune surveillance, cell debris clearance and resolution of local inflammation

A common clinical observation with advanced cancer patients is the differential responses to systemic treatments where some metastatic lesions may be less or more responsive to therapy compared to lesions located at other anatomical sites. Such frequently encountered clinical phenomenon strongly suggests that the local TME plays crucial roles in modulating therapeutic responses. For example, in a retrospective study of 371 metastatic melanoma patients treated with the first FDA-approved “modern” immunotherapy high-dose IL-2, the response rate in patients with cutaneous or subcutaneous metastasis was ~ 50% whereas with visceral metastases it was only 13%; more strikingly, in individual patients harboring both cutaneous/subcutaneous and visceral metastases, tumor regression took place only at cutaneous/subcutaneous lesions whereas visceral metastases progressed upon the same systemic IL-2 therapy [25]. Furthermore, in more recent retrospective studies exploring the relationship between metastases and anti-PD-1 immunotherapy in melanoma and non-small cell lung cancer (NSCLC) patients, it was discovered that the presence or absence of liver metastases was significantly associated with reduced objective response rate (ORR, 30.6% vs. 56.3%) and shortened median progression-free survival (mPFS, 5.1 vs. 20.1 months) in melanoma, and was related to a significant difference of progression-free survival (mPFS 1.8 vs. 4.0 months) in NSCLC as well [26, 27].

Despite the clear relevance derived from multiple clinical studies, further in-depth mechanistic investigations that require procuring clinical specimens from multiple metastatic organ sites can be logistically challenging. To that end, an exceptional case study reported a single patient with high-grade serous ovarian cancer who was treated with multiple chemotherapy regimens and exhibited regression of some metastatic lesions with concomitant progression of other lesions [28]. After comprehensive biological profiling of the metastatic lesions, it was found that while the aggressively progressing metastases were characterized by immune cell exclusion, the regressing and stable metastases were heavily infiltrated by CD8+ and CD4+ T cells and exhibited oligoclonal expansion of specific T cell subsets. This study was a rare but direct examination of clinical samples that unequivocally demonstrated the crucial role of distinct tumor immune microenvironments co-existing within an individual patient and impacting the heterogeneous responses of metastatic lesions to a homogenous systemic therapy [28].

To broadly elucidate the underlying biological mechanisms of the organ specific TME impact on immunotherapy responses, multiple studies have used preclinical murine models harboring metastases at multiple anatomical sites and having treatments by various immunotherapy agents. For example, implanting murine 4T1 mammary cancer cells at subcutaneous or intratibial sites and FACS profiling of isolated tumor-associated immune cells revealed significant differences in the immune composition (macrophages, dendritic cells, CD8+ and CD4+ T cells, etc.) depending on the sites of tumor growth [29]. Additionally, responses to an immunotherapy treatment regimen consisting of three agonist antibodies, Tri-mAb (anti-DR5, anti-CD40 and anti-4-1BB) were compared among multiple pairs of subcutaneous and orthotopic cancer models (renal, colon or prostate) [30]. It was observed that orthotopically implanted tumor lesions responded significantly less to therapy than the same tumor type located subcutaneously. Reimplantation experiments confirmed that tissue specific TME was the determinant of differential responses to therapy. Compared with subcutaneous tumors, orthotopic tumors had a prominent type 2 immuno-suppressive microenvironment. More importantly, causal factors were identified and neutralizing the macrophage- and Th2-associated molecules, e.g. chemokine CCL2 and cytokine IL-13, significantly improved therapeutic responsiveness [30]. Similarly, tissue immune microenvironments were shown to be determinants of differential responses to immunotherapy treatments in other murine models, such as a study using the syngeneic CT26 cells to compare orthotopic colon cancer and subcutaneous cancer lesions [31] and another study using the syngeneic B16F10 murine melanoma cells to compare subcutaneous and lung tumor lesions [32].

Unfortunately, despite fair amount of effort by the research community to compare differential anatomic responses to cancer therapies, the brain, one common metastasis organ site, has been adversely neglected [33, 34]. With better management of systemic diseases and prolonged survival, brain metastasis incidence has kept increasing in multiple cancer types in recent years [35, 36]. Almost all clinical trials exclude patients with CNS metastasis in fear of multiple factors, such as the brain’s impermeability to therapeutic agents, and rapid deterioration of patients’ status caused by CNS progression [37, 38]. In the following sections of this review, we will discuss the immuno-biology in the CNS microenvironment and its impact on immunotherapy efficacy.

## Immuno-biology of the brain microenvironment

While the primary regulatory and cognitive functions of the central nervous system (CNS) are conducted by neuronal circuitry, it is also essential to maintain a homeostatic environment of stable metabolism and inflammation, which tasks fall on the large number of stromal cells in the brain, including astrocytes and microglia cells [39]. To that end, the CNS is shielded from the rest of blood circulation by the presence of blood–brain barrier (BBB) [40] and establishes its own homeostatic regulatory system distinct from other organs in the body [41]. Brain is generally regarded as an immune privileged sanctuary organ site, where immune responses must be tightly regulated to prevent overwhelming and potentially damaging immune reactions [42]. The latest evidence suggests that cells in the brain constantly and actively regulate immune responses; and dysregulation of such regulation may contribute to the pathogenesis of neurodegenerative diseases [43], malignant transformation of glioma [44], and outgrowth of metastatic tumors [45]. Thus, understanding immuno-biology of the distinctive metabolic and inflammatory microenvironment in the CNS is a prerequisite for successful immunotherapy targeting brain metastases.

Intracranial malignant neoplasms can arise either from primary brain tumors (mainly glioma) or from secondary cancers metastatic to the CNS. Lung cancer, breast cancer and melanoma are the major sources of brain metastases, which collectively outnumber primary malignant brain tumors by ~ 10:1 [46, 47]. While both gliomas and CNS metastases may share the same anatomical site of tumor growth, similar treatment modalities (surgery, radiation and chemotherapy), devastating impact on quality of life and dismal prognosis, the underlying disease pathogenesis and interaction between tumor cells and shared TME may be vastly different. In primary brain malignancies such as glioblastoma (GBM), up to 30–40% of the tumor mass can be composed of resident and infiltrating myeloid cells [48–50], while tumor-infiltrating lymphocytes (TIL) represents a tiny portion (< 0.25%) of cells isolated from human GBM biopsies [51]. In contrast, close examination of host reactions in murine models of breast cancer brain metastasis demonstrated that host astroglial and microglial cells became drastically activated and accumulated around metastatic cells shortly after tumor cell extravasation upon carotid artery injection, suggesting very early involvement of brain defense mechanism during the metastatic process [52]. The cytokine signaling axis CXCR4/CXCL12 may facilitate the brain metastasis as it was shown to promote the cancer cell adhesion and migration through the brain endothelial vasculature [53, 54]. A postmortem investigation of 17 human tissue specimens of established brain metastases (derived from melanoma, breast and lung cancers) revealed profound activation and heterogenous distribution of microglial cells surrounding the established brain macro-metastatic lesions [55]. The microglial cells exert multiple physiological functions in the brain metastasis microenvironment that include antigen presentation, phagocytosis, and direct cytotoxicity through nitric oxide and superoxide expressions [56], as well as interaction with and neurotoxic activation of astrocytes [57]. To that end, our group reported characterization of intricate relationships among breast cancer, astroglial and microglial cells where astrocyte-released exosomes transfer PTEN-targeting microRNA into cancer cells to mediate PTEN down-regulation in the cancer cells, which ultimately results in CCL2 up-regulation and recruitment of brain metastasis-promoting monocytes [58].

Intriguingly, infiltration with adaptive immune T lymphocytes is highly heterogenous in brain metastasis lesions, varying from total absence to very dense infiltration [55, 59]. A study of 116 brain metastasis specimens using immunohistochemistry revealed that not only the density of TIL but also the composition of TIL subtypes differ among individual patients. Properties of the primary cancer cells (e.g. tumor mutation burden and neoantigen load) likely contribute to the adaptive immune response to brain metastasis as the TIL density from melanoma-derived brain metastasis is significantly higher than that from other tumor types. Importantly, patients with brain metastases who present with dense infiltration of effector CD3+, cytotoxic CD8+, or memory CD45RO+ TILs showed a significantly favorable survival prognosis compared to patients with little or absent TIL infiltration [59]. Immune escape has been increasingly recognized as a universal hallmark of cancer and as the mechanism to evade and overcome immune surveillance [60, 61]. Therefore, it is not surprising that infiltration of immune suppressive FOXP3+ regulatory TILs, as well as exhausted PD-1+ TILs, has been observed in the majority of these brain metastasis specimens [59]. Consistently, in a study comparing NSCLC-derived brain metastases and matched primary tumors, PD-L1 expression was more frequently observed in brain metastases than in the matched primary tumors [62].

Lastly, how do adaptive immune T cells infiltrate brain metastatic lesions in the presence of blood–brain barrier (BBB)? Pharmacodynamic studies in preclinical murine models of breast cancer brain metastasis showed that BBB permeability could be compromised, and vascular leakiness became highly heterogeneous depending on the progression of brain metastatic outgrowth [63]. Furthermore, a prominent adverse event of CAR-T immunotherapy is cerebral edema (neurotoxicity) caused by massive T cell infiltration into the brain parenchyma [64]. Though the exact mechanism for this severe adverse phenotype remains elusive, it is clear from pathological examination that the BBB is significantly disrupted in these adverse cases [65]. Therefore, the distinctive vascular properties of the CNS and its remodeling under disease conditions also partially contribute to the immuno-biology of brain metastasis.

## Response of brain metastasis to immunotherapy and strategies to enhance efficacy

Brain is conventionally regarded a major organ site of metastasis with sanctuary immune status; hence, up till recently, most clinical trials exclude patients with CNS metastases [37, 38]. With the advent of immunotherapies, particularly the immune checkpoint blockade (ICB), the efficacy of immunotherapy in the context of brain metastasis has been understudied in the clinic; yet it remains an emergent medical need and substantial clinical interest has developed whether ICB could be as effective in managing brain metastasis as its remarkable efficacy in controlling extracranial metastases. The intracranial activity of immunotherapy was first noted in the post hoc analysis of a phase III clinical trial comparing single agent or combination of the anti-CTLA-4 ipilimumab or gp100 peptide vaccine in metastatic melanoma patients (NCT00094653) [17]. This initial signal led to an open-label phase II study of single-agent ipilimumab for patients with melanoma-derived brain metastases [66], which showed modest intracranial activity. At the same time, despite exclusion of patients with CNS metastases in all of the early clinical trials targeting another immune checkpoint PD-1/PD-L1 pathway, a single-center phase II study of anti-PD-1 pembrolizumab in patients with melanoma and NSCLC-derived brain metastases showed promising results [67].

Based on these clinical studies showing early signals of positive intracranial activity, a landmark trial of treating melanoma brain metastasis with ICB combination of anti-PD-1 nivolumab and anti-CTLA-4 ipilimumab has been conducted (CheckMate 204, NCT02320058) [68]. It was an open-label phase II study of patients with untreated melanoma brain metastasis, which led to remarkable clinical efficacy showing intracranial clinical benefit rate (primary end point) of 57% among the 94 patients being evaluated.

**Fig. 1:**
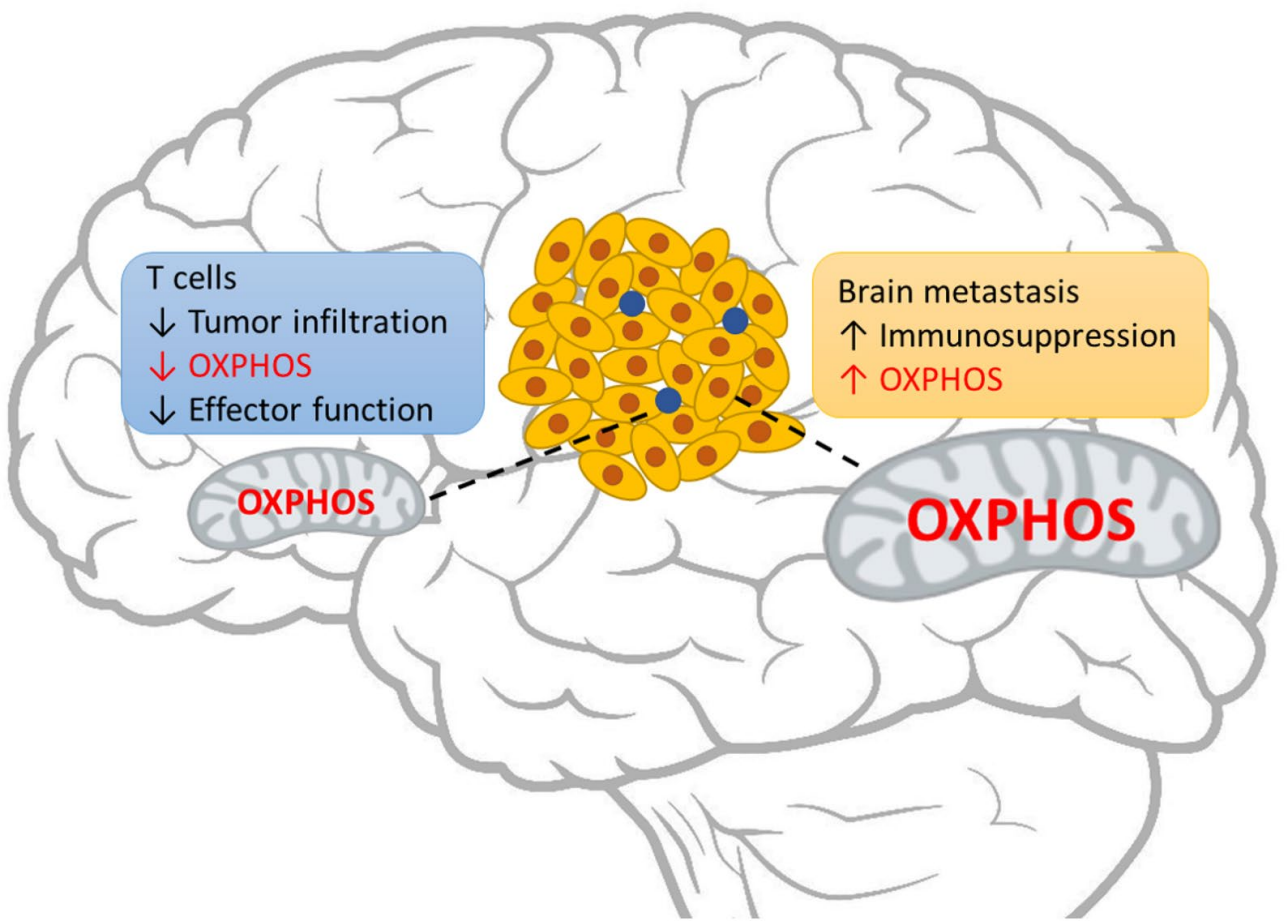
Brain-specific metabolic TME may contribute to immunotherapy efficacy. In the mammalian CNS, mitochondrion-dependent oxidative phosphorylation (OXPHOS) is a favorable mechanism of cellular metabolism. Examination of clinical specimens suggests an immunosuppressive TME in the brain with fewer T cell infiltration, which is dependent on OXPHOS for effector functions, and elevated levels of OXPHOS activities in brain metastases. This highlights a prominent example where brain specific TME metabolic environment may contribute to therapeutic response

Despite these promising results, the advances are still in the early stage and most patients with CNS metastases remain difficult to treat; the benefit must also be extended to brain metastases from other primary cancer types in addition to melanoma. Additionally, the underlying mechanism of resistance versus response of the intracranial tumors to systemically administered ICB remains a fundamentally unanswered question. Because immune cells are highly dynamic in response to physiological alterations, their biological functions must be tightly coupled to the cellular metabolism that provides the underlying material and energetic support. From in vitro studies, it was believed that activation of T cells shifts cellular metabolism towards glycolysis which takes place in the cytosol and produces more biological building blocks in preparation of cell proliferation and clonal expansion, and that naïve and memory T cells are more dependent on mitochondrion-dependent oxidative phosphorylation (OXPHOS) which yields more ATP to confer higher spare respiratory capacity [69]. However, a recent study showed that in vivo isolated TIL cells, whose immune effector functions are impaired, gradually lose OXPHOS activity by progressive loss of PPAR-gamma coactivator 1α (PGC1α), a master transcriptional regulator of mitochondrial biogenesis, and that metabolic reprogramming of OXPHOS through forced expression of PGC1α restores and enhances the T cell cytolytic activities [70]. Additionally, while OXPHOS is differentially regulated in M1 or M2 macrophages, the T lymphocytes do not shut down OXPHOS during activation but instead significantly remodel the mitochondrial proteome [71, 72]. Therefore, mitochondria and its dynamics, which includes opposing fusion and fission processes, have appeared to exert profound influence on effective immunity [73, 74]. At the center of mitochondrial functions, OXPHOS is conducted through five multi-subunit protein complexes lining in the cristae membranes. Individually, these distinct protein complexes also participate in various inflammatory regulations. For example, reactive oxygen species (ROS) are potent mediators of inflammation, which are mainly produced by complexes I and III of the OXPHOS chain [75]. Further, succinate is a metabolite with important inflammatory signaling functions, whose conversion to fumarate is mediated by succinate dehydrogenase, complex II of the OXPHOS cascade [76]. Given the unique metabolic microenvironment in the brain [77], it is reasonable to speculate that brain specific metabolic pathway may modulate response to ICB therapy. Along this line, in another recent study that compared patient-matched melanoma brain metastases and extracranial tumor lesions, it was found that the brain metastatic lesions have significantly lower T cell infiltration (immunosuppressive) and higher OXPHOS activity than those from extracranial tumor specimens. Further, such differences were replicated in multiple human melanoma xenograft models where cancer cells were implanted subcutaneously and intracranially and compared with RNA-seq expression profiling. Importantly, treatment with an in-pipeline OXPHOS complex I inhibitor significantly improved animal survival in multiple preclinical melanoma brain metastasis models [78]. Collectively, these findings suggest that in the brain TME, the metastatic tumor cells are more dependent on mitochondrion mediated OXPHOS metabolism. Since immune cells are also dependent on OXPHOS metabolism for cytolytic effector functions [70], increased OXPHOS metabolic activity in tumor cells may deprive necessary nutrient substrates for infiltrating immune cells and result in an immunosuppressive TME in the CNS metastatic lesions (Fig. 1). It would be clinically impactful to determine whether the increased OXPHOS activity of brain metastatic tumors may causally confer resistance to ICB immunotherapy; and whether overcoming such intracranial metabolic dysregulation by combinatorial targeting of brain specific metabolic pathway and immune checkpoints may further enhance the efficacy of existing immunotherapy.

## Conclusions and future direction

Currently, immunotherapy benefits a small number of patients across multiple cancer types, and the immuno-biological microenvironments at distinct metastatic organ sites are important determinants for differences in efficacy. In particular, the local immuno-biological TME at metastatic organ sites, partly mediated by the tissue-resident innate immune cells, interacts with the systemic adaptive immune system to determine responses to immunotherapy. Therefore, the major goal of future efforts should be deepening the understanding of underlying immuno-biological mechanisms responsible for the organ-specific anti-tumor immune responses and based on such knowledge developing strategies to expand the benefits of immunotherapy to more patients with advanced metastatic cancer. To that end, it is important to analyze clinical specimens of immunotherapy treated metastases of all cancer types (responders and non-responders, early relapses and long-term survivors) to examine immune cell infiltration and composition, as well as local stromal cell alterations, which will reveal novel insight into the response mechanism of immunotherapy in distinctive metastatic TMEs. Additionally, it is necessary to perform mechanistic studies and functional validations using appropriate animal models in order to better understand the immuno-biology underlying effective immunotherapy.

In the CNS, it has been convincingly established, by retrospective examinations of brain metastasis specimens and prospective clinical studies of ICB treatment of patients with brain metastases, that in some patients the brain can harbor an “active” immune microenvironment that respond to immunotherapy. Enrichment of mitochondrion mediated OXPHOS is a unique metabolic trait of brain metastasis; modulating the OXPHOS activity in the brain TME may impact efficacy of ICB treatments. To overcome the immunosuppressive TME in the CNS, future efforts should also include combining checkpoint inhibitors with other treatments (radiation, chemotherapy, targeted therapy, oncolytic viruses, etc.). The rapid progress in clinical investigations and preclinical studies will pave the way for effective modulation of the brain metastasis tumor microenvironment that allows effective T cell-mediated responses and enables more brain metastasis patients to benefit from immunotherapy.

In summary, persistent endeavor in clinical investigations and preclinical studies to explore effective ways of manipulating the local immuno-biological microenvironment will likely enhance and expand the efficacy of immunotherapy to more metastatic diseases.

## Data Availability

Not applicable.

## Abbreviations

ACT: adoptive cell transfer
BBB: blood–brain barrier
CAR-T: chimeric antigen receptor T cell
CNS: central nervous system
GBM: glioblastoma
ICB: immune checkpoint blockade
mPFS: median progression-free survival
NSCLC: non-small cell lung cancer
ORR: objective response rate
OXPHOS: oxidative phosphorylation
ROS: reactive oxygen species
TAM: tumor-associated macrophage
TIL: tumor-infiltrating lymphocyte
TME: tumor microenvironment
